# Altered systemic bile acid homeostasis contributes to liver disease in pediatric patients with intestinal failure

**DOI:** 10.1038/srep39264

**Published:** 2016-12-15

**Authors:** Yong-Tao Xiao, Yi Cao, Ke-Jun Zhou, Li-Na Lu, Wei Cai

**Affiliations:** 1 Department of Pediatric Surgery, Xin Hua Hospital, School of Medicine, Shanghai Jiao Tong University, Shanghai, China; 2 Shanghai Institute for Pediatric Research, Shanghai, China; 3 Shanghai Key Laboratory of Pediatric Gastroenterology and Nutrition, Shanghai, China

## Abstract

Intestinal failure (IF)-associated liver disease (IFALD), as a major complication, contributes to significant morbidity in pediatric IF patients. However, the pathogenesis of IFALD is still uncertain. We here investigate the roles of bile acid (BA) dysmetabolism in the unclear pathogenesis of IFALD. It found that the histological evidence of pediatric IF patients exhibited liver injury, which was characterized by liver bile duct proliferation, inflammatory infiltration, hepatocyte apoptosis and different stages of fibrosis. The BA compositions were altered in serum and liver of pediatric IF patients, as reflected by a primary BA dominant composition. In IF patients, the serum FGF19 levels decreased significantly, and were conversely correlated with ileal inflammation grades (r = −0.50, p < 0.05). In ileum, the inflammation grades were inversely associated with farnesoid X receptor (FXR) expression (r = −0.55, p < 0.05). In liver, the expression of induction of the rate-limiting enzyme in bile salt synthesis, cytochrome P450 7a1 (CYP7A1) increased evidently. In conclusion, ileum inflammation decreases FXR expression corresponding to reduce serum FGF19 concentration, along with increased hepatic bile acid synthesis, leading to liver damages in IF patients.

Pediatric intestinal failure (IF), as a result of short bowel syndrome (SBS) or gastrointestinal motility disorders, is a condition characterized by insufficient bowel function to maintain hydration and nutrient absorption for growth and development^1^,^2^. IF-associated liver disease (IFALD) is a serious complication and the leading cause of morbidity and mortality in children IF patients^3^,^4^. However, the mechanisms underlying the development of IFALD are poorly understood. To unravel the mechanisms of IFALD, we performed population based cross-sectional study on serum fibroblast growth factor 19 (FGF19) and bile acid (BA) homeostasis in relation to histological liver damage in pediatric IF patients.

The human FGF family includes about 23 members with diverse biological functions^5^. FGF19 is secreted by ileum in response to activation of farnesoid X receptor (FXR) with bile acids^6^,^7^. FGF19 has been shown to regulate BA homeostasis through a negative feedback control of bile salt synthesis in human^8^. FGF19 has also been implicated in the regulation of carbohydrate, lipid and energy metabolism in the liver^9^,^10^. The studies recently have been demonstrated that the decreased serum concentration of FGF19 associated with increased BA synthesis in patients with Crohn’s disease and intestinal failure^11^,^12^,^13^. Annika *et al*. reported that total or partial loss of ileum decreased serum FGF19 concentration corresponding to hepatic inflammation and fibrosis^12^. However, the roles of FXR/FGF19 signaling in BA metabolism among pediatric IF patients remained unknown. We hypothesized that pediatric IF patients decreased serum concentration of FGF19 in association with ileal inflammation, and corresponding to bile acid dysmetabolism, leading to histological liver injury. To this end, we determined the serum concentrations of FGF19, pro-inflammatory cytokines, histological liver injury, biochemical liver function tests and serum BA levels, and analysis the relationship between them.

## Results

### IF patients characteristics

A total of twenty-three patients at median age 8.0 months (IQR 3.3–58.7) participated in the study (Table 1). Causes of IF included short bowel syndrome (necrotizing enterocolitis (NEC): n = 4, small bowel atresia: n = 5, and mid-gut volvulus: n = 3) and intestinal dysmotility disorders (chronic intestinal pseudo-obstruction (CIPO): n = 7 and extensive aganglionosis of hirschsprung’s disease: n = 4). No significant differences in age were observed in controls and IF patients. In total, twenty patients preserved Ileocaecal valve and ileum. Five patients were on parenteral nutrition (PN) and eighteen had weaned off PN 0.6 years (0.5–0.9) earlier, after 3 months (1.7–4) on PN. The PN energy comprised 50.79% (47.14–55.36) of glucose and 31.96% (28.03–36.25) of fat. PN fat was given as soy oil-based emulsion [1.5 g/kg/day (1.10–1.78)] and combined with fish oil-based emulsion (0.8 g/kg/day) in two patients.

### The IF patients exhibit histological liver injury

**Figure 1:**
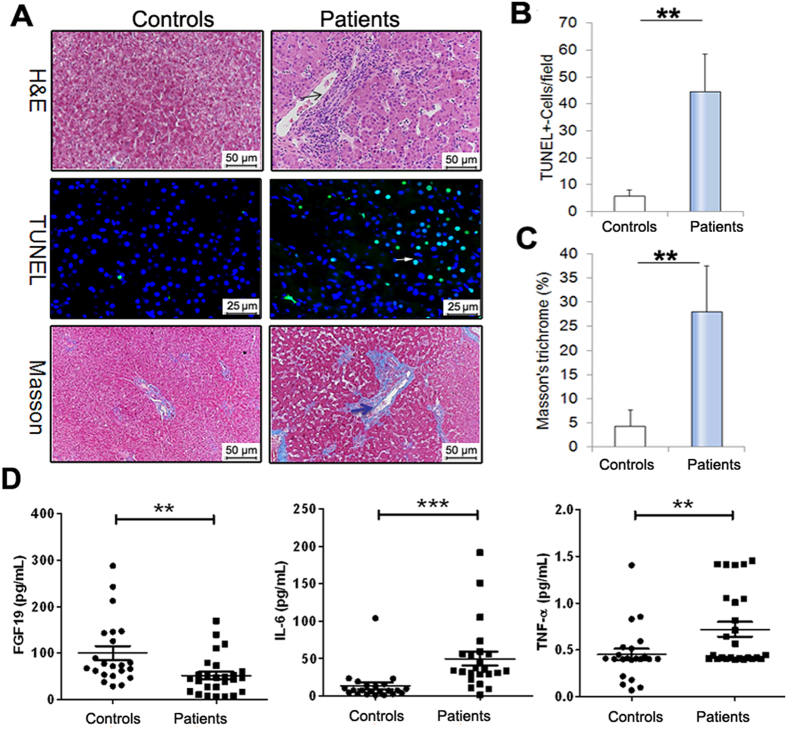
Liver histological changes in pediatric IF patients. (**A**) Pediatric IF patients exhibit inflammatory infiltrate (H&E staining, n = 9), hepatic apoptosis (TUNEL-stained, n = 7) and fibrosis (Masson trichrome assay, n = 7). (**B**) Quantification of TUNEL-positive cells. (**C**) Quantification of Masson trichrome analysis. (**D**) Serum FGF19 concentration was significantly lower in pediatric IF patients compared to controls. Conversely, serum levels of IL-6 and TNF-α were significantly higher in patients than in controls. Scale bar = 25 or 50 μm. **p < 0.01; ***p < 0.001.

As seen in Fig. 1A, the control liver tissues exhibited normal liver histology. In contrast, the liver sections from pediatric IF patients exhibited liver damages characterized by bile duct proliferation, lymphocytes infiltration, and hepatocyte ballooning (Fig. 1A). Apoptotic hepatocytes were detected by TUNEL staining of liver sections. As expected, few TUNEL-positive cells were observed in the control liver specimens. Conversely, TUNEL-positive hepatocytes increased significantly in liver sections from patients (Fig. 1A and B). When compared with the controls, extensive portal fibrosis was indicated in liver slides of pediatric IF patients (Fig. 1A and C).

### The FGF19 is associated with liver injury and inflammation

As shown in Fig. 1D, in IF patients, serum FGF19 concentration was significantly lower (n = 23, 51.42 ± 47.03 pg/mL, p < 0.01) compared to controls (n = 21, 102.31 ± 71.23 pg/mL) (Fig. 1D). As seen in Table 2, abnormal values in liver enzymes, and parameters of cholestasis were indicated. The values of liver enzymes including alkaline phosphatase (ALP) (51.76 ± 69.24 U/L vs. 90.34 ± 66.99 U/L), alanine aminotransferase (ALT) (23.23 ± 16.01 U/L vs. 55.77 ± 19.91 U/L, p < 0.01) and aspartate aminotransferase (AST) (36.35 ± 16.66 U/L vs. 61.67 ± 27.51 U/L, p < 0.01) were increased in patients’ serum compared to the controls (Table 2). Plasma total bilirubin (5.33 ± 2.55 μmol/L vs. 6.24 ± 1.35 μmol/L) and conjugated bilirubin (3.16 ± 1.43 μmol/L vs. 3.19 ± 2.26 μmol/L) were unregulated in patients that compared to controls (Table 2). Correlated analysis showed that serum FGF19 concentrations were inversely correlated with both the levels of ALP (r = −0.34, p = 0.03) and AST(r = −0.3, p = 0.06) (Table 2). As indicated in Fig. 1D, we also found that the pro-inflammatory factors serum interleukin-6 (IL-6) (13.28 ± 21.78 pg/ml vs. 50.63 ± 45.99 pg/ml, p < 0.001) and tumor necrosis factor-alpha (TNF-α) (0.41 ± 0.20 pg/ml vs. 0.89 ± 0.43 pg/ml, p < 0.01) concentrations were higher in patients compared to controls (Fig. 1D and Table 2). In addition, the serum IL-6 (r = −0.32, p < 0.05) was inversely associated with serum FGF19 (Table 2).

### FXR/FGF19 signaling contributes to BA homeostasis

**Figure 2:**
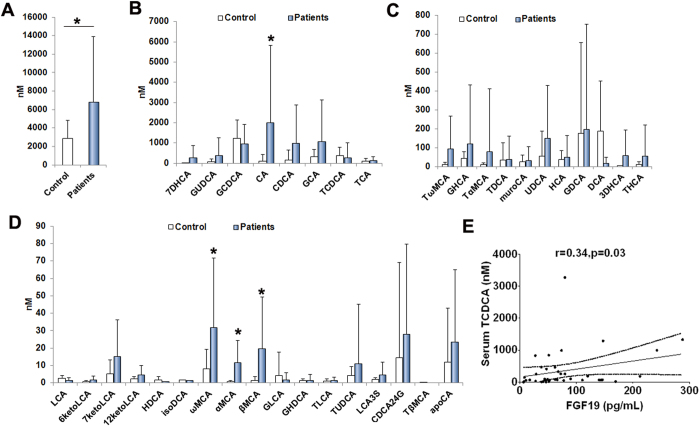
Altered levels of blood bile acids composition in IF patients. (**A**–**D**) The total and composition of BA in plasma were markedly altered in IF patients with a significant in the proportion of the primary BA but decreased proportions of the secondary/tertiary BA (Control, n = 18, Patients, n = 21). (**E**) Serum FGF19 levels significantly positively related to concentration of TCDCA. *p < 0.05.

**Figure 3:**
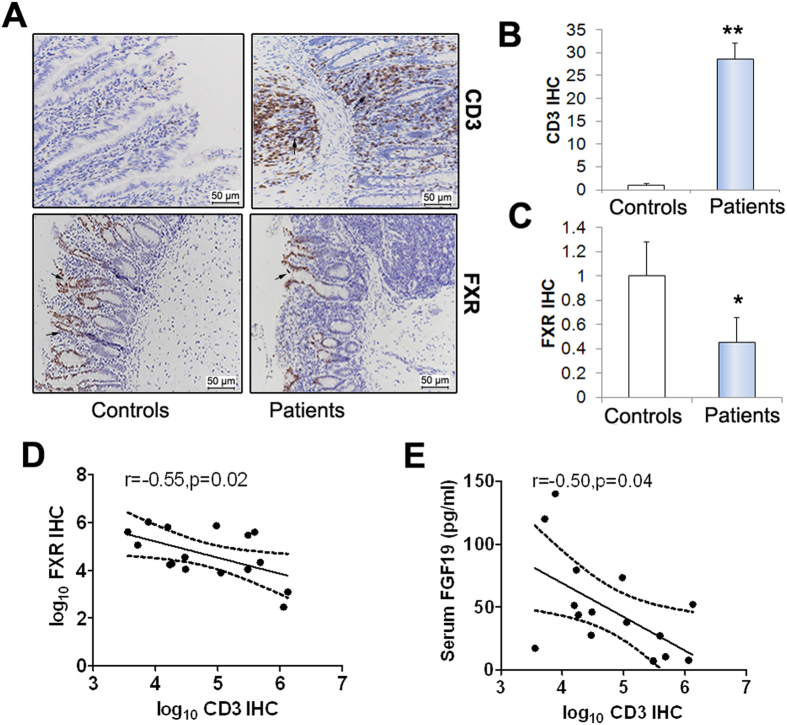
Pediatric IF patients exhibit impaired activation of intestinal FXR/FGF19 axis. (**A**) Immunohistochemical (IHC) analysis for CD3 and FXR in ileal tissues from patients (n = 7) and controls (n = 6). (**B**,**C**) Quantification of CD3 IHC and FXR IHC. (**D**,**E**) CD3 IHC conversely correlated with FXR IHC and serum FGF19 concentrations. Scale bar = 50 μm. *p < 0.05; **p < 0.01.

As shown in Fig. 2A, the total plasma BA significantly elevated in IF patients (Fig. 2A). The BA composition of plasma was markedly altered in pediatric patients with a significant in the proportion of the primary bile acid, including the cholic acid (CA) and α, β, ω-muricholic acid (MCA) (Fig. 2B–D). In contrast, the secondary or tertiary bile acids, including the deoxycholic acid (DCA), taurochenodeoxycholic acid (TCDCA), lithocholic acid (LCA) and hyodeoxycholic acid (HDCA), slightly decreased in IF patients compared to controls (Fig. 2B–D). As a FXR agonist, TCDCA positively related to concentration of serum FGF19 (Fig. 2E). In intestinal, we found that the IF patients without intestinal surgery, suffering from ileal inflammation, had numbers of CD3-positive lymphocytes infiltrating ileal mucosa (Fig. 3A and B). As result of ileal mucosa injury, the FXR expression was reduced significantly in patients when compared to controls (Fig. 3A and C). As shown in Fig. 3D, the CD3-positive lymphocytes in ileal mucosa negatively correlated to the FXR expression (Fig. 3D). In addition, ileal mucosa inflammatory degrees was inversely associated with serum FGF19 levels (r = −0.5, p = 0.04) (Fig. 3E).

**Figure 4:**
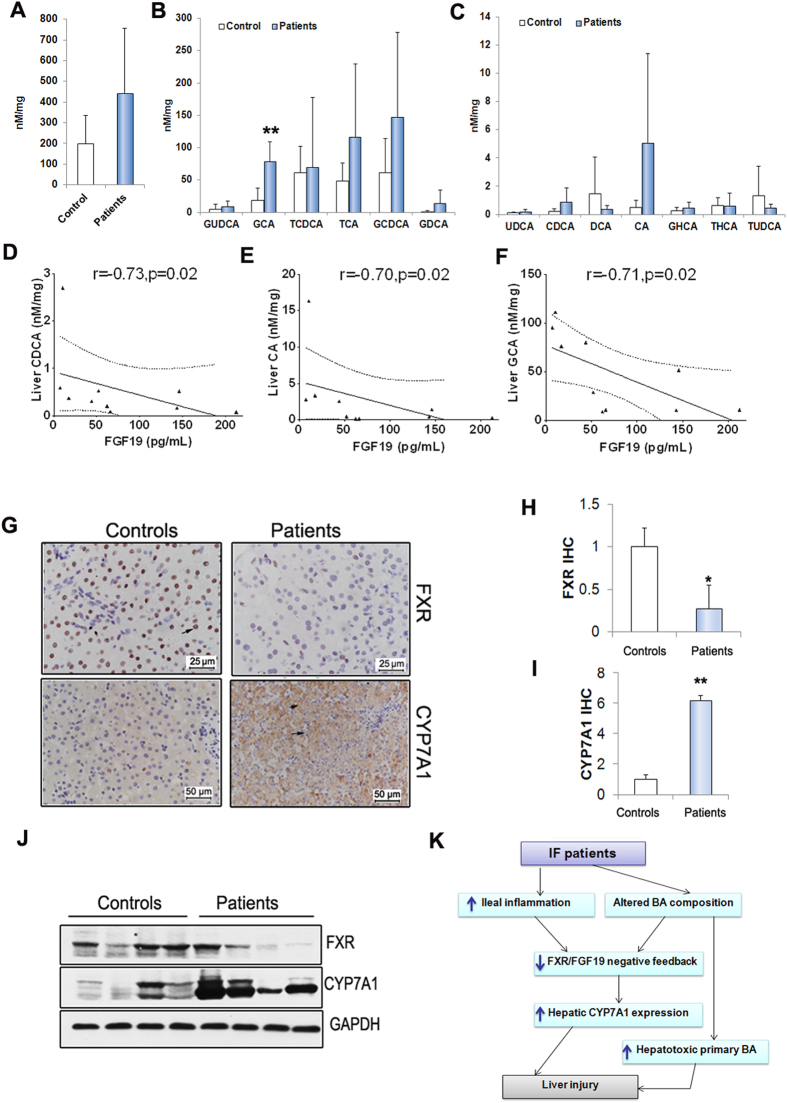
Decreased FGF19 impaired CYP7A1 expression in liver. (**A**–**C**) Altered BA composition in liver tissues. (**D**–**F**) Serum FGF19 levels inversely correlated with primary bile acids including CA, CDCA and GCA in liver (Control, n = 5, Patients, n = 5) (**G**) FXR and CYP7A1 IHC. (**H**,**I**) Quantification of panel G. (**J**) Western-blot analysis for FXR and CYP7A1. (**K**) The scheme illustrates a potential mechanism by which FGF19 contributes to development of IFALD. Scale bar = 50 μm. *p < 0.01.

As seen in Fig. 4A, the total BA contents in IF patients’ liver were higher (442.49 ± 312.93 nmol/mg) compared to controls (199.15 ± 134.59 nmol/mg) (Fig. 4A). The BA composition of the liver tissues of patients was also altered when compared with controls. Both unconjugated and conjugated primary BA, including cholic acid (CA), glycocholic acid (GCA), taurocholic acid (TCA), chenodeoxycholic acid (CDCA) and glycodeoxycholic acid (GDCA), increased in livers of patients related to the controls (Fig. 4B and C). As seen in Fig. 4D–F, contents of CA (r = −0.70, p < 0.05), CDCA (r = −0.73, p < 0.05) and GCA (r = −0.71, p < 0.05) inversely correlated with serum FGF19 (Fig. 4D–F). FXR is an important BA receptor and essential to the BA homeostasis^14^,^15^,^16^. We here showed that the hepatic FXR expression was lower in IF patients compared to controls (Fig. 4G–J). In liver, the classic bile acid synthesis is controlled by cholesterol 7a-hydroxylase (CYP7A1)^17^. We here found that the hepatic CYP7A1 protein expression increased significantly in patients when compared to the controls (Fig. 4G–J).

## Discussion

In this study, we firstly showed that pediatric IF patients exhibited liver injury that characterized by cholestasis, portal inflammation, as well as hepatic apoptosis and fibrosis. Secondly, It demonstrated that the altered FXR/FGF19 signaling was contributed to the cholestasis and liver injury in pediatric IF patients.

As alteration in BA composition can cause hepatotoxicity^18^, thus analysis of the BA composition is essential to assess the impact of altered BA composition on the development of IFALD. In this study, the IF patients exhibited increased levels of primary BA in blood and liver, including CA and CDCA, concurrent with decreased levels of secondary or tertiary BA, such as DCA. In human, the primary BA, including CA and CDCA, can converted into secondary BA, DCA, LCA, through gut microbial 7-dehydroxylation^19^. The primary BA increased in IF patients might be attributed to differences in gut microbiota composition that shifts the microbial modification of the BA. Similar changes in BA composition levels have been reported in various liver diseases, including cirrhosis^20^, alcoholic liver disease^21^, cholestasis^22^, in which it was observed increased CDCA levels. In addition, alterations in BA metabolism are likely to induce the liver damage through affecting the solubilization of phospholipids, cholesterol and other lipids^23^. It reported that pro-inflammatory cytokines, including IL-6 and TNF-α, had been shown to be important mediators of cholestatic liver injury^24^,^25^. We here found that serum IL-6 and TNF-α concentrations were higher in IF patients compared to controls, which suggest alterations of bile acids metabolism potentiate hepatotoxicity may partly through pro-inflammatory mechanisms.

During bile acids enterohepatic circulation, FGF19 mediates bile acids hemostasis through a negative feedback way^9^,^26^. In the enterocyte, bile acids reclaimed by terminal ileum can upregulate FGF19 gene expression via activation of FXR^27^. After releasing into circulation, FGF19 reaches the liver and inhibits hepatic bile acids synthesis through suppression of CYP7A1 gene that encodes the rate limiting enzyme in synthesis of bile acids^28^. In this study, serum FGF19 concentrations were markedly decreased in IF patients compared to healthy controls. Annika and colleagues recently showed that loss of ileum led to reduced FGF19 production in patients with intestinal failure^12^. However, the mechanisms underlying the FGF19 associated with liver injury is still not fully established. Although patients with ileum preserved in this study, the inflammatory infiltrating presented in ileum, resulted in intestinal mucosa injury and further reduced FXR expression. In addition, we also indicated serum FGF19 concentrations were inversely associated with pro-inflammatory cytokine IL-6. IL-6 has been previously reported to involved in cholestasis among infants and children with intrahepatic and extrahepatic cholestasis^29^. The intestinal injury in IF patients could cause terminal ileum epithelial cells damaged and reduced the terminal ileum FXR expression. In contrast, the intestinal injury may allow translocation of bacterial antigens in the circulation and cause increased expression of pro-inflammatory including IL-6, leading to disturbed hepatocyte bile acid homeostasis. These findings here support the hypothesis that ileal inflammation leads to impaired intestinal FGF19 expression and altered bile acid homeostasis may through inhibiting ileal FXR activation in patients with IF. Experimental study indicated that administration of FGF19 protected the liver from cholestasis by reducing hepatic synthesis and primary hydrophobic BA^30^. In our study, we showed that serum FGF19 levels inversely correlated with hepatic primary bile acids including CA, CDCA and GCA. In addition, the expression of CYP7A1 was markedly increased in liver tissues from patients compared controls, which reinforce the concept that FGF19 can protect liver from cholestatic liver injury via repression of CYP7A1 expression, which suggests that FGF19 may be an important mediator in the pathogenesis of IFALD. Indeed, we showed that FGF19 were conversely related to the markers of liver injury, such as alkaline phosphatase, (ALP). Interestingly, we here did not found that FGF19 was tightly correlated to the bilirubin or lipids in the serum.

As pediatric IF is a rare disease, a relatively limited sample has been one of several limitations in this study. Although these results of our study show association rather than causality, this study adds novel data on FXR/FGF19 pathway in the pathogenesis of IF in pediatric patients. Further studies conducted in more patients with biopsy-proven steatosis, portal inflammation, and histological cholestasis or with different fibrosis stages are warranted to analysis relationship between them and FGF19. Collectively, these results bring new insight to possible approaches for prevention and treatment of IF.

## Materials and Methods

### Patients

This study was approved by the Faculty of Medicine’s Ethics Committee of Xin Hua hospital (XHEC-C-2016-063), School of Medicine, Shanghai Jiao Tong University, Shanghai, China. A total of 23 serum samples and 7 liver specimens were obtained from pediatric IF patients who underwent surgery. A total of 21 serum specimens were obtained from healthy controls with matched age. 6 normal adjacent non-tumour tissues, taken from hepatoblastoma patients, were used as liver controls. All patients’ guardians provided written informed consent. The patients’ characteristics are presented in Table 1. All methods in this study were carried out in accordance with the relevant guidelines.

### Biochemical measurements in serum

A total of 21 controls’ serum samples and 23 patients’ serum samples were analyzed in this study. The blood samples were analyzed for alanine aminotransferase (ALT), aspartate aminotransferase (AST), and bilirubin and conjugated bilirubin by using routine hospital laboratory methods. Serum total cholesterol, triglycerides, and high density lipoprotein cholesterol (HDL-C) and low density lipoprotein cholesterol (LDL-C) were determined enzymatically.

### Determination of FGF19, IL-6 and TNF-α

Serum samples were stored at −80 °C until analyzed after blood collection and centrifugation. FGF19, IL-6 and TNF-α concentrations in serum were measured by ELISA kits (R&D Systems, MN, USA) according to the manufacturer’ s instructions (21 controls and 23 patients).

### Histological analyses and fibrosis determination

Histological examination was stained with hematoxylin and eosin (H&E). Fibrosis was determined by mason’s trichrome stain according to the method described in a previous study^31^. Masson’s trichrome staining was performed according to the manufacturer’s protocol (Genmed Scientifics, Wilmington, DE). The collagen fiber was stained blue, the nuclei were stained black, and the background was stained red. Liver tissues were analyzed for apoptosis using the TUNEL test (TdT-mediated dUTP nick end labeling) was performed using the “*In Situ* Cell Death Detection Kit” from Roche Diagnostics according to the manufacturers’ instructions. Sections were analyzed with a fluorescence microscope. Liver biopsies were analyzed by two researchers and a pathologist, blinded to clinical data, until consensus was reached.

### Western-blot and Immunohistochemistry (IHC)

Western-blot and Immunohistochemistry (IHC) assays were performed as previously described^32^. For western-blot, the protein was extracted from liver tissues of IF patients using RIPA buffer with protease inhibitor cocktail (Pierce). The soluble fraction of the cell lysates was isolated by centrifugation at 12, 000 g for 10 minutes in a microfuge. BCA reagent (Pierce, Rockford, IL, USA) was applied to determine protein concentration. The equal amounts of proteins (150 ug/well) were separated by 4–12% SDS-PAGE, and transferred to nitrocellulose membranes. The membranes were incubated overnight at 4 °C with primary antibodies. Antibodies for GAPDH (Cell signaling technology, Danvers, MA, USA), CYP7A1 (Millipore, Darmstadt, Germany) and FXR (Invitrogen, Camarillo, CA) were used here. After incubation, the membranes were washed with PBS (containing 0.1% Tween) and incubated with horseradish-peroxidase conjugated detected the antigen-antibody complexes using an ECL Plus chemiluminescence reagent kit (Pierce, Rockford, IL, USA). For immunohistochemistry (IHC) analysis, paraffin-embedded tissues were incubated with xylol and descending concentrations of ethanol. Antigen retrieval was performed using citrate buffer, pH 6.0 or PH 8.0. Endogenous peroxidases were removed by incubation with 0.3% H_2_O_2_ for 15 minutes at room temperature (RT) and blocking was performed using 10% bovine serum albumin (BSA) for 1 hour at RT. Primary antibodies were then applied in an optimal concentration overnight in a wet chamber (CYP7A1. Millipore, Darmstadt, Germany; CD3, Abcam, Bristol, UK and FXR, Invitrogen, Camarillo, CA). Following incubation overnight at 4 °C, the slides were rinsed in phosphate-buffered saline (PBS) and incubated with the secondary antibody for 1 hour at RT. Antibody binding was visualized by a liquid DAB Substrate Chromogen System (Dako, Glostrup, Denmark). The slides were rinsed in PBS and counterstained with hematoxylin. IHC images analysis was used software Image Pro Plus (Media Cybernetics) 20 fields/sample.

### Measurement of bile acid composition

Bile acid composition was determined according to the methods that previously reported^33^,^34^. A total of 40 bile acids including cholic acid (CA), glycocholic acid (GCA), taurocholic acid (TCA), chenodeoxycholic acid (CDCA), glycochenodeoxycholic acid (GCDCA), taurochenodeoxycholic acid (TCDCA), deoxycholic acid (DCA), glycodeoxycholic acid (GDCA), taurodeoxycholic acid (TDCA), ursodeoxycholic acid (UDCA), glycoursodeoxycholic acid (GUDCA), tauroursodeoxycholic acid (TUDCA), lithocholic acid (LCA), glycolithocholic acid (GLCA), taurolithocholic acid (TLCA), hyocholic acid (HCA), glycohyocholic acid (GHCA), taurohyocholic acid (THCA), α-muricholic acid (αMCA), tauro-α-muricholic acid (TαMCA), β-muricholic acid (βMCA), tauro- β-muricholic acid (TβMCA), ω-muricholic acid (ωMCA), tauro-ω-muricholic acid (TωMCA), hyodeoxycholic acid (HDCA), glycohyodeoxycholic acid (GHDCA), taurohyodeoxycholic acid (THDCA), murocholic acid (MuroCA), dehydrocholic acid (DHCA), glycodehydrocholic acid (GDHCA), taurodehydrocholic acid (TDHCA), 3-dehydrocholic acid (3-DHCA), 7-dehydrocholic acid (7-DHCA), isodeoxycholic acid (isoDCA), apocholic acid (apoCA), 6-ketolithocholic acid (6-KLCA), 7-ketolithocholic acid (7-KLCA), 12-ketolithocholic acid (12-KLCA), 23-nordeoxycholic acid (23norDCA), and dehydrolithocholic acid (DHLCA). Deuterated internal standards (IS) lithocholic acid-2,2,4,4-D4 (LCA-D4) and cholic acid-2,2,4,4-D4 (CA-D4) obtained from Steraloid, Inc. (Newport, RI) as standards. A total 39 plasma samples (18 controls and 21 patients) and 10 liver samples (5 controls and 5 samples) were analyzed in this study. Briefly, about 50 μL of plasma was added 150 μL of methanol with IS (100 nmol/L of CA-D4 and LCA-D4), and then the mixture was incubated for 10 minutes at RT. After centrifugation at 20 000 g for 10 min, 160 μL of supernatant was transferred to a clean tube. The supernatant was dried under vacuum and reconstituted with 40 μL of acetonitrile (with 0.1% formic acid) and 40 μL of water (with 0.1% formic acid). After centrifugation, 5 μL of supernatant was injected for measurement. Liver tissue was weighed and homogenized in 50 μL of icecold 40% methanol, and then centrifuged at 20 000 g for 10 min. The supernatant was transferred to a clean tube. Then, 80 μL of ice-cold methanol/chloroform (3:1, v/v) was added to the remaining pellet and rehomogenized. After centrifugation, the two supernatants were combined, spiked with 10 μL of IS (1200 nmol/L of CA-D4 and LCA-D4), and dried under vacuum. The extracts were reconstituted with 40 μL of acetonitrile (with 0.1% formic acid) and 40 μL of water (with 0.1% formic acid). After centrifugation, 5 μL of supernatant was injected for measurement. The analysis was performed with a Waters ACQUITY ultra performance liquid chromatography (BEHC-18, 1.7 μm 2.1 × 100 mm column) coupled with Waters Xevo TQ-S triple quadrupole mass spectrometry (Waters MS Technologies, Ltd.). Data acquisition and bile acids quantification were performed using the MassLynx 4.1 software (Waters, Ltd.).

### Statistical analysis

Data statistics are presented as medians IQR or mean ± SD. For comparisons of different groups, statistical significance was determined based on the Student’s t test. Correlations were tested with Spearman rank correlation. P values < 0.05 were considered statistically significant.

## Additional Information

**How to cite this article**: Xiao, Y.-T. *et al*. Altered systemic bile acid homeostasis contributes to liver disease in pediatric patients with intestinal failure. *Sci. Rep.* **6**, 39264; doi: 10.1038/srep39264 (2016).

**Publisher’s note:** Springer Nature remains neutral with regard to jurisdictional claims in published maps and institutional affiliations.

## Figures and Tables

**Table 1:** Patients characteristics.

Variable	Median (IQR)
Patients (n)	23
Male/Female (n)	16/7
Age (months)	8 (3.3–58.7)
Gestation age (weeks)	39 (38–40)
Gestation weight (g)	3200 (2800–3568)
Weight Z-score	−4.2 (−7.3~−3.2)
Height Z-score	−3.3(−4.4~−1.6)
Age at PN start (months)	4.5 (1.6–11.3)
Duration of PN (months)	3.9 (2–6)
Weaned off PN (n)	13
Time after weaning off PN (years)	0.6 (0.5–0.9)

Data are frequency or median (interquartile range, IQR). PN, parenteral nutrition.

**Table 2:** Liver biochemistry, serum lipids, glucose and inflammatory cytokines in the patients.

Variable	Control	Patients	p value	Correlation with FGF19	
	n = 21	n = 23	Control vs. patients	r	p value
**Liver enzymes**					
Plasma alkaline phosphatase, ALP (U/L)	51.76 ± 69.24	90.34 ± 66.99	0.08	−0.34	0.03
Plasma alanine aminotransferase, ALT (U/L)	23.23 ± 16.01	55.77 ± 19.91	<0.01	−0.19	0.24
Plasma aspartate aminotransferase, AST (U/L)	36.35 ± 16.66	61.67 ± 27.51	<0.01	−0.3	0.06
**Markers of cholestasis**					
Plasma total bilirubin (μmol/L)	5.33 ± 2.55	6.24 ± 1.35	0.15	−0.15	0.37
Plasma conjugated bilirubin (μmol/L)	3.16 ± 1.43	3.19 ± 2.26	0.97	−0.07	0.67
**Serum lipids**					
Serum HDL cholesterol (mmol/L)	0.76 ± 0.26	0.63 ± 0.19	0.09	0.07	0.64
Serum LDL cholesterol (mmol/L)	1.50 ± 0.56	1.83 ± 0.45	0.04	0.02	0.92
Serum total cholesterol, TC (mmol/L)	2.00 ± 0.47	2.4 ± 0.61	0.02	−0.16	0.32
Serum triglycerides, TG (mmol/L)	0.79 ± 0.31	1.13 ± 0.60	0.03	0.08	0.6
Plasma glucose (mmol/L)	3.80 ± 1.16	3.21 ± 0.88	0.21	0.18	0.26
**Markers of inflammation**					
Serum IL-6 (pg/mL)	13.28 ± 21.78	50.63 ± 45.99	<0.01	−0.32	0.04
Serum TNF-α (pg/mL)	0.41 ± 0.2	0.89 ± 0.43	<0.01	−0.33	0.32
